# The use of a lot quality assurance sampling methodology to assess and manage primary health interventions in conflict-affected West Darfur, Sudan

**DOI:** 10.1186/s12963-016-0103-3

**Published:** 2016-10-06

**Authors:** Kiemanh Pham, Emily Chambers Sharpe, William M. Weiss, Alexander Vu

**Affiliations:** 1 Department of Emergency Medicine, Johns Hopkins School of Medicine, Baltimore, MD USA; 2 Department of International Health, Center for Refugee and Disaster Response, Johns Hopkins Bloomberg School of Public Health, Baltimore, MD USA; 3 Global Health, HIV/AIDS, and Nutrition Consultant, Charlotte, NC USA

**Keywords:** Lot quality assurance sampling, Monitoring and evaluation, Conflict, Complex humanitarian emergency

## Abstract

**Background:**

Organizations working in conflict-affected areas have a need to monitor and evaluate their programs, however this is often difficult due to the logistical challenges of conflict areas. Lot quality assurance sampling may be a suitable method of assessing programs in these situations.

**Methods:**

We conducted a secondary data analysis of information collected during Medair’s routine program management functions. Medair’s service area in West Darfur, Sudan was divided into seven supervisory areas. Using the available population information, a sampling frame was developed and interviews were conducted from randomly selected caretakers of children in each supervisory area every six months over 19 months. A survey instrument with questions related to key indicators for immunizations and maternal, newborn, and child health was used for the interviews. Based on Medair’s goals for each indicator, decision rules were calculated for the indicators; these decision rules determined which supervisory areas and indicators performed adequately in each assessment period. Pearson’s chi-squared tests, adjusted for the survey design using STATA “svy: tab” commands, were used to detect overall differences in coverage in this analysis.

**Results:**

The coverage of tetanus toxoid vaccination among pregnant women increased from 47.2 to 69.7 % (*p* value = 0.046), and births attended by a skilled health professional increased from 35.7 to 52.7 % (*p* value = 0.025) from the first to last assessment periods. Measles vaccinations declined from 72.0 to 54.1 % (*p* value = 0.046). The estimated coverage for the proportion of women receiving a postpartum dose of vitamin A (54.7 to 61.3 %, *p* value = 0.44); pregnant women receiving a clean delivery kit (54.6 to 47.1 %, *p* value = 0.49); and pentavalent vaccinations (49.7 to 42.1 %, *p* value = 0.28) did not significantly change.

**Conclusions:**

Lot quality assurance sampling was a feasible method for Medair staff to evaluate and optimize primary health programs in a conflict-affected area. Medair managers were able to collect, analyze, and disseminate data to staff alongside the routine work of the organization. These results suggest LQAS may be used in other complex humanitarian emergencies in which there are logistical challenges and limited resources.

## Background

In 2013, the Organisation for Economic Co-operation and Development estimated that industrialized countries donated $134.8 billion USD in aid to developing countries [1]. Donors are increasingly requiring funded organizations to not only deliver high quality services but also conduct rigorous monitoring and evaluation programs to assess the impact of these service [2]. Independent of donor requirements, organizations implementing programs want frequently updated information to help their own managers optimize services to meet organizational targets and make positive changes for beneficiaries.

Since active conflict began in the Darfur region of Sudan in 2003 [3], Sudan has been a major recipient of international humanitarian assistance. In 2012 it received the ninth-largest amount of humanitarian aid, and the total amount of assistance from the United States alone stands at $887 million USD [4]. Despite the amount of aid that is directed towards Sudan, it has been challenging for non-governmental organizations (NGOs) to provide services, particularly in the Darfur region where much violence has occurred [5]. The ongoing, protracted conflict in Darfur has progressively decreased the humanitarian space, making the delivery and monitoring of aid services difficult [6]. After the International Criminal Court charged Sudanese president Omar al-Bashir with war crimes and crimes against humanity in 2009, al-Bashir expelled ten international NGOs working in Darfur [7, 8]. Furthermore, armed militia have specifically directed attacks at aid organizations by looting supplies and kidnapping aid workers [9]. Adding to these difficulties, there are logistical challenges with the delivery of services in Darfur, since large distances separate communities across areas that are subject to violence [10]. In response, the United Nations World Food Programme (WFP) established a helicopter service to ferry aid groups to service sites [11]. The flight schedule set by the WFP, in combination with security measures limiting humanitarian workers’ ability to stay overnight in field locations, regularly restricts project managers’ time in communities to 2–3 h, hampering the ability of aid workers to monitor and maintain programs.

### Description of Medair’s programs in West Darfur

From 2001 to 2012, the Switzerland-based NGO Medair worked in West Darfur [12], a state with an estimated population of 1.3 million [13] and one of the three states that comprise the Darfur region. Medair’s humanitarian assistance programs in West Darfur included primary healthcare service provision and maintenance of health clinics; health promotion activities; support for the Sudanese Ministry of Health programs; and water, sanitation, and hygiene service provision and promotion. These programs were conducted in both refugee and internally displaced persons (IDP) camps, as well as in towns with sizeable refugee and IDP populations, serving a total population of approximately 325,000. As part of their primary healthcare programs, Medair provided supportive supervision, quality assurance, and finance and human resources for primary health care workers – including midwives – who worked in West Darfur Ministry of Health clinics and primary health care workers who worked in clinics located in refugee and IDP camps. Medair also provided logistic and human resource support and intensive health promotion campaigns to augment the immunization programs conducted by the West Darfur Ministry of Health.

### Description of the Medair/Johns Hopkins Monitoring and Evaluation Program

In 2010, Medair collaborated with the researchers from the Johns Hopkins School of Medicine and Bloomberg School of Public Health to establish a monitoring and evaluation (M&E) program to track indicators focused on primary healthcare coverage in West Darfur. The M&E program was initiated in the context of Medair having to expand its programs after the 2009 expulsion of ten NGOs working in Darfur. One of Medair’s goals was to have population-based methods of measuring the effectiveness of primary health care services in West Darfur. At the same time, Medair wanted to give its program managers on the ground a tool to empirically guide the management of primary health care programs.

Given the multiple challenges of logistics and security in West Darfur, a management method using lot quality assurance sampling (LQAS) was proposed for the monitoring and evaluation of Medair’s services. LQAS was originally developed in the 1920s as a method of assuring a minimum level of quality in industrial production [14]. Using LQAS concepts, a health program manager decides three factors which determine the sampling plan: (1) a target percent coverage for an indicator (the upper threshold); (2) a percent coverage below which a lot is considered unacceptable (the lower threshold); and (3) the tolerable levels of misclassifying failures and successes (the alpha and beta errors, respectively) [15, 16]. The three factors yield a decision rule; if a lot sample has fewer successes than the decision rule, the lot is classified as having unacceptable quality.

Valadez and colleagues have published a protocol for LQAS in health programs, which simplifies LQAS procedures [17]. In their formulation, LQAS is typically carried out in two stages. In the first stage, a random sample of 19 is taken within several local supervision areas (SA), which are usually subsets of a larger program catchment area. Supervision areas are assessed for performance of key health indicators; if the number of successes in an SA is greater than the pre-determined decision rule, then the performance of the relevant health indicator is classified as acceptable. The protocol simplifies LQAS by setting the sample size at 19 and a 30 % difference in the upper and lower thresholds, resulting in less than a 10 % probability of misclassification (i.e., the alpha and beta errors are less than 10 %). In the second stage, information from several supervision areas are combined and weighted, and an estimate for the entire program area is made for each health indicator. In certain sampling designs, the estimates for the program area are comparable in accuracy to estimates made from typical 30-cluster sample surveys that are often carried out by NGOs in low- and middle-income countries [18]. LQAS provides additional value over cluster sampling by providing information about the performance of health indicators within the program area (by supervision area), while cluster sampling provides estimates at the overall program catchment area level only. LQAS therefore allows program managers to identify variation in performance among supervision areas and to prioritize problem-solving in SAs with inadequate performance, rather than assuming performance is the same across the program area. In addition, the survey work for LQAS can be organized at the local level of each SA, and can be carried out concurrent with program activities using the same logistics (transport, personnel, etc.). As a result, programming does not need to stop for a period to allow for data collection activities, and survey costs can be shared with the routine costs of program activities.

LQAS has been used to assess many development programs globally [15] (such as malaria [19], malnutrition [18], and immunization [20]). There are few studies, however, that discuss the feasibility of using LQAS for measuring health indicators and outcomes in conflict-affected settings. Deitchler, et al. compared the precision, time, and cost of different variations of cluster surveys in West Darfur with a secondary aim of using LQAS hypothesis tests with each variation [21]. In another paper, Valadez and colleagues describe the use of LQAS in South Sudan to assess maternal and newborn health indicators [22].

The many advantages of LQAS made it preferable to the cluster survey method for Medair in West Darfur, where there are multiple challenges including the distance between the communities served by Medair and the tenuous security situation. Medair managers and supervisors had limited access to the field, and felt that the ability to conduct LQAS alongside usual programmatic work was a particular advantage of LQAS over cluster surveys. The objective of this paper is to describe the process of using the LQAS method in a conflict-affected region as a process to evaluate the effectiveness of primary health programs, and to guide the management of these programs.

## Methods

This is a secondary data analysis of information collected during routing program management, monitoring, and evaluation of the Medair program. The monitoring and evaluation of primary health programs was carried out from May 2010 through December 2011 in West Darfur, Sudan. Four separate LQAS assessments were conducted, allowing measurement of key indicators approximately every six months. Medair and a group from Johns Hopkins had agreed to collaborate during these four assessments with the ultimate goal of training Medair staff such that Medair could independently continue assessments after this period without additional technical assistance.

### Development of the sampling plan

**Fig. 1:**
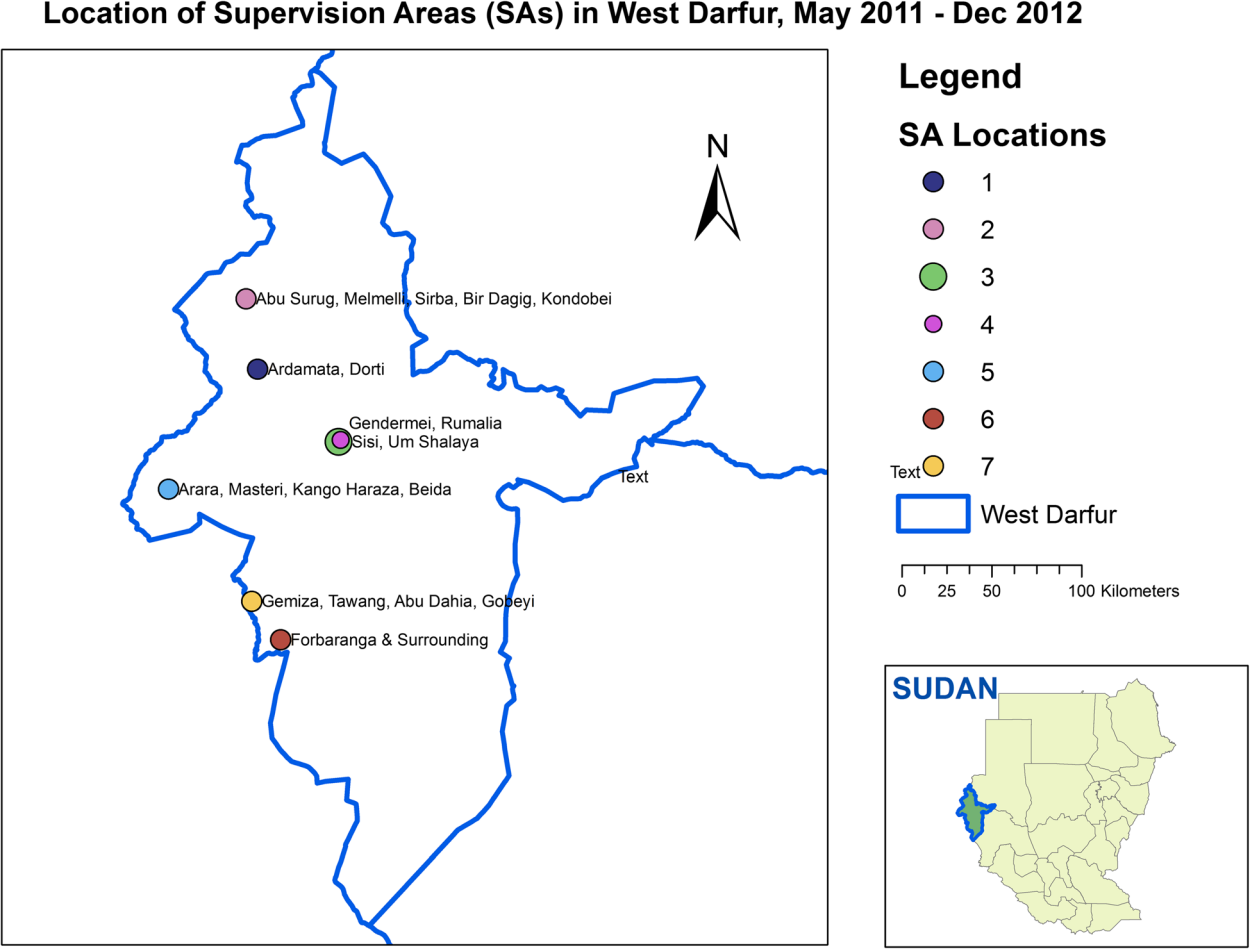
Map of Medair’s program areas in West Darfur, Sudan

As an initial step of random sampling, the Medair service area was divided into seven supervisory areas. The SAs were determined based both on geography and on Medair’s organizational structure, such that one manager could be assigned to each SA (Fig. 1). The SAs also corresponded with Medair’s seven areas for supervised and supported primary health care clinics and health promotion activities.

A sampling plan requires estimates of the population in the geographic area of interest, however true population estimates in Darfur are difficult to obtain [23]. Even without conflict, migration is common among the Darfur population; conflict added further instability to settlements and communities, making the determination of true population numbers a challenge [23]. For Medair’s LQAS program, the Sudanese government ministries and the United Nations organizations operating in the catchment area agreed upon the estimated population figures. Where estimated population information was not available or incomplete, we asked other NGOs for their population estimates of the communities. We then calculated the cumulative population of the serviced community (Table 1). For a given set of questions, nineteen households were selected in each SA, with the probability of a community being selected proportional to the community’s population [24]. We assumed that the proportion of women 15–49 years of age with live births in the 2 years prior to the survey (the target population of Medair’s services) was relatively constant in the SAs. For each round of LQAS, changes to the population listing were made as needed, and a new set of 19 households were selected in each SA.

**Table 1:** Supervisory areas and population^a^

Region	Supervisory area (SA)	Population estimates	Community type
El Geneina	1	34,960	IDP Camps
Northwest	2	49,110	Towns & IDP Camps
East 1	3	29,505	Towns & IDP Camps
East 2	4	55,633	IDP Camps
Southwest	5	60,623	Towns & IDP Camps
Forboranga Town	6	50,885	Towns & IDP Camps
Forboranga/Habila Rural	7	44,626	Towns & IDP Camps

Note: ^a^These population figures refer to communities Medair serviced, not to West Darfur as a whole. The 2008 population of West Darfur was estimated at 1.3 million by the Sudanese government

### Selection of indicators and development of the survey

With input from the Medair staff, portions of the United Nations Children’s Fund’s (UNICEF) Multiple Indicator Cluster Survey 4 (MICS4) questionnaire were adapted for our assessment [25]. Specifically, we adapted questions that provided information on the following indicators: immunization coverage for diphtheria, pertussis, and tetanus (indicator 3.3); measles immunization coverage (indicator 3.4); hepatitis B immunization coverage (indicator 3.5); neonatal tetanus protection (indicator 3.7); and skilled attendant at delivery (indicator 5.7). In 2011, the World Health Organization no longer recommended vitamin A supplementation in postpartum women [26]. However, Medair continued this practice. In order to monitor Medair’s programs, an indicator for vitamin A supplementation in postpartum mothers from the MICS3 was also included [27]. Finally, the number of women who had received a clean delivery kit prior to their last delivery was also measured because Medair managers felt it was important to assess this indicator, even though it is not a part of the MICS3 or MICS4. The 2010 Sudan Household Health Survey (SHHS), a countrywide assessment of public health indicators conducted by the Sudanese government, was based on the MICS4 [28]. The use of questions from MICS4 thus enabled the comparison of key indicators between areas where Medair provided primary health services to areas where Medair did not work.

The adapted questionnaire was translated from English to Sudanese Arabic, the language of study participants. The questionnaire was then back-translated by local translators; back-translations were reviewed by the research team and the local Medair team working in West Darfur. This process was necessary even though an Arabic translation of the MICS4 is available from UNICEF because the target population often did not understand the wording of UNICEF’s Arabic translation. Additional edits were made through an iterative process to ensure that the translation captured the essence of each question. Pilot testing was done with convenience samples in two communities of IDPs. Interviewers made notes of the capacity of the target population to understand and respond to the questionnaire. The results of the pilot testing were reviewed jointly by Johns Hopkins investigators and local Medair team members, and final edits were made based on the feedback from interviewers.

### Participant recruitment and data collection

The majority of communities could only be accessed by helicopter, and the time in those communities was often restricted to two to three hours. In the first LQAS round, the Medair staff developed a detailed logistic plan that included delineating which staff members were going to specific communities with exact times for flight manifests. Medair staff worked on the survey separate from their usual work during the first round of data collection so that they could concentrate on learning data collection and management methods without the distraction of their usual work duties. During subsequent LQAS rounds, Medair staff working in each locality conducted interviews with participants under the supervision of research coordinators. To leverage the advantages of the LQAS methodology, survey work in these later rounds was done alongside the routine tasks of Medair staff on days when the staff visited assigned program communities that overlapped with the LQAS sample selection. The completed questionnaires were transported by helicopter or car to the main Medair office in El Geneina, the capital of West Darfur, for data entry and analysis.

Once staff arrived in a community, they conferred with a community leader or other key informant to develop a map of the community. Our team also informed community leaders about our planned activities and the purpose of the interviews, and we asked for any input community leaders had as a means of engaging the population. From these discussions, the community was divided into subsections, from which a subsection was randomly selected using a random number table. If the subsection had more than approximately 30 households, it was further divided and the process of random selection was repeated until an area was chosen with approximately 30 households to facilitate the process of randomly selecting a household [17]. The households were then numbered, and one was randomly chosen using a random number table. For large communities where more than one sampling unit was located, the process of selecting a household was repeated.

A parallel sampling method was used to administer the questionnaire [29]. In each randomly selected household, women between the ages of 15 and 49 who had a live birth in the 2 years prior to the interview were enrolled. If a household did not have a woman who met the inclusion criteria, surveyors went to the next nearest household as measured from the front door of one dwelling to the front door of the next. Questions in the survey were grouped depending on the age group of children to which they referred. For example, immunization questions required children that were at least 1 year of age while questions on MNCH indicators referred to children younger than 1 year. Thus, staff asked appropriate question groups depending on the age of children in a household. If a qualifying household did not have children from the age group required for a group of questions, the next closest households were screened until children from each age group were included in the survey. As a result, more than 19 participant households were required in each SA to complete the different set of questions. Verbal consent was obtained from all participants from a standardized consent script that was read to the participants by the surveyors. Verbal consent was used instead of written consent to ensure the respondents were not placed at an increased risk of harassment from local militias for participating in the study as would be the case if a written document were in the household’s possession.

Medair managers selected staff members to be trained in LQAS survey techniques, which took place over a three-day period of class work and one day of hands-on practice in a nearby community. Staff who showed a particular aptitude for the survey techniques were designated as survey supervisors. During the survey, supervisors oversaw the household selection process and reviewed completed paper questionnaires to assure completeness prior to leaving the community. Medair staff entered responses from the paper questionnaire into a database that was created in Epi Info (version 3.5.4, Centers for Disease Control and Prevention, Atlanta, GA).

### Statistical analysis

For each of the randomly selected 19 households in each SA, a number of positive responses to questions about a health indicator (“successes”) were used to classify whether or not a health indicator’s performance in that SA was adequate. The classification was done by comparing the number of positive responses to a pre-determined decision rule value that had been calculated using LQAS sampling rules [17]. If the number of positive responses did not exceed the decision rule value, the performance of the health indicator in an SA was classified as inadequate; otherwise, the performance of the indicator was classified as adequate. Next, the responses from all SAs in the program area were pooled. A population estimate (including a mean and 95 % confidence interval) for the total program area was calculated for each indicator using the population estimates of each SA to weight the data.

For the first round of LQAS, the pooled percentage (unweighted) for each indicator was used along with LQAS sampling rules to determine the decision rule to be used in each SA. Doing so allowed the project to classify SAs as either adequate or inadequate for each indicator, as determined by baseline figures. For subsequent assessments, Medair managers established pre-determined decision rules for each indicator by using data from previous rounds of LQAS as well as Medair’s organizational goals and the managers’ projections of the capacity of the SAs to improve based on programmatic intervention.

The data were then imported into Stata/SE (version 11, StataCorp, College Station, TX) for analysis at the program level using Stata’s ‘svy’ options. Stata allowed us to estimate the performance of each indicator (with mean and 95 % confidence interval) by each round. We weighted the data by the population size of each SA and adjusted the standard errors for clustering at the SA level. To assess the overall change in Medair’s programs during the assessment period, we used Pearson’s chi-squared tests, using STATA “svy: tab” commands to adjust for the survey design to determine if there was a statistically significant difference (*p* value < .05) for each indicator using the same population weights and clustering adjustments.

Before starting surveillance, approval was obtained from the Johns Hopkins School of Medicine Institutional Review Board and the Sudanese Government Humanitarian Aid Commission at the national level in Khartoum and at the state level in West Darfur.

## Results

### Demographic characteristics of the sample

A total of 1323 women participated across the four rounds of LQAS assessments from May 2010 to December 2011 (Table 2). The average ages of participants in the four rounds were similar, with a range in mean age of 26.5 to 27.6. The most common level of education was no schooling, with approximately two-thirds of women reporting no formal education. The second most common level of education was a primary education, which 21.1–24.4 % of women reported throughout the assessment period. Almost all of the women were married (a range of 93.1–99.4 %), and the average number of children the participants reported was just over two (2.1–2.2).

**Table 2:** Demographic Characteristics of Study Participants

	Assessment 1 (May 2010)	Assessment 2 (Oct. 2010)	Assessment 3 (May 2011)	Assessment 4 (Dec. 2011)
Total interviewed	315	338	348	322
Age (Years)				
Mean	27.6	26.5	27.4	26.5
Standard Deviation	7.9	5.8	7.4	6.6
Education level (%)				
No schooling	64.5	68.6	66.1	68.9
Primary	21.7	22.9	24.4	21.1
Intermediate	0.3	0.0	1.4	0.6
Secondary	2.2	2.0	1.2	1.9
University/Higher Institute	0.6	0.0	0.3	0.0
Khalwa (Religious School)	11.0	7.5	4.0	5.6
Adult Education	(Not recorded)	(Not recorded)	2.6	1.9
Married (%)	93.1	97.6	98.3	99.4
Number of children				
Mean	2.2	2.2	2.2	2.1
Standard Deviation	1.3	0.9	1.1	1.0

### LQAS evaluation results

Several indicators for maternal, newborn, and child health showed improvement from the first to last LQAS rounds when calculated at the program level (Tables 3 and 4). Tetanus toxoid vaccination coverage for neonates increased throughout the assessment period from 47.2 to 69.7 % (*p* value = 0.046). In the last two rounds of LQAS, all the SAs were classified as adequate for this indicator. The only time an SA was classified as inadequate for tetanus toxoid coverage was SA7 during the first round. The coverage of births attended by a skilled health professional also improved significantly from 35.7 % in the first round to 52.7 % in the final round of LQAS (*p* value = 0.025, Table 3). At most, one SA was classified as inadequate in each assessment period, and in the final round of LQAS, all SAs were classified as adequate.

**Table 3:** Maternal and Child Health Indicators

Indicators	Protection against neonatal tetanus				Births attended by a skilled health professional			
Assessment periods	May 2010	Oct. 2010	May 2011	Dec. 2011	May 2010	Oct. 2010	May 2011	Dec. 2011
Target Coverage	50 %	50 %	60 %	60 %	35 %	30 %	45 %	45 %
2010 Sudan Household Health Survey Result for West Darfur [28]	46.5 %				33.4 %			
Decision Rule	7	7	9	9	4	3	6	6
Program Coverage (95 % CI)	47.2 % (28.4, 66.8)	53.8 % (48.1, 59.3)	76.9 % (59.3, 88.4)	69.7 % (54.9, 69.5)	35.7 % (21.0, 53.6)	34.5 % (22.2, 49.4)	40.2 % (24.1, 58.6)	52.7 % (39.0, 66.0)
*P* value for difference between 1^st^ & 4^th^ Rounds	0.046				0.025			
Number of Successes								
SA1	13	11	17	16^c^	8	12	10	15^c^
SA2	^a^	9	14	14	^a^	5	8	12
SA3	11	12	15	15	6	2^*^	6	11
SA4	7	11	16	14	3^*^	4	6	7
SA5	10	9	18	12	10	9	13	12
SA6	12	11	11	11	9	6	6	8
SA7	2^*^	9^b^	11	13	4	7^d^	3^*^	7

Notes: *SAs that do not reach the benchmark. ^a^Due to insecurity, samples were not taken from SA2 in the May 2010 round of LQAS. ^b^18 samples instead of the usual 19 were obtained; the decision rule is unchanged. ^c^20 samples instead of the usual 19 were obtained; the decision rule is unchanged. ^d^18 samples instead of the usual 19 were obtained; the new decision rule is 2

**Table 4:** Maternal and Child Health Indicators

Indicators	Women who received a clean delivery kit				Mothers receiving 1 dose of postpartum vitamin A			
Assessment periods	May 2010	Oct. 2010	May 2011	Dec. 2011	May 2010	Oct. 2010	May 2011	Dec. 2011
Target Coverage	50 %	35 %	55 %	55 %	50 %	45 %	60 %	60 %
2010 Sudan Household Health Survey Result for West Darfur [28]	Not Reported				15.3 %			
Decision Rule	7	4	8	8	7	6	9	9
Program Coverage (95 % CI)	54.6 % (30.5, 76.7)	63.8 % (50.3, 75.4)	56.8 % (38.9, 73.1)	47.1 % (33.7, 60.9)	54.7 % (39.2, 69.3)	54.7 % (40.0, 68.6)	45.5 % (30.6, 61.3)	61.3 % (48.9, 72.4)
*P* value for difference between 1^st^ & 4^th^ Rounds	0.49				0.44			
Number of Successes								
SA1	12	13^b^	15	16^d^	8	15	10	7^*d^
SA2	^a^	7	9	12	^a^	8	6^*^	11
SA3	9	8	7^*^	10	14	9	15	11
SA4	7	5	10^c^	11	10	9	13	6^*^
SA5	14	7	9	8	9	7	13	6^*c^
SA6	2^*^	4	3^*^	6^*^	9	5^*^	9	4^*c^
SA7	8	6^b^	5^*^	10	3^*^	7^c^	7^*^	8^*c^

Notes: *SAs that did not reach the benchmark. ^a^Due to insecurity, samples were not taken from SA2 in the May 2010 round of LQAS. ^b^18 samples instead of the usual 19 were obtained; the new decision rule is 3. ^c^18 samples instead of the usual 19 were obtained; the decision rule is unchanged. ^d^20 samples instead of the usual 19 were obtained; the decision rule is unchanged

Two other MNCH indicators did not improve (Table 4). The coverage of women who received a clean delivery kit did not significantly change between the initial and final rounds of LQAS (from 54.6 to 47.1 %, *p* value = 0.49). For this indicator, results during the May 2011 round of LQAS were particularly poor as three of the seven SAs were classified as inadequate. SA6 was the poorest performing among all the SAs since it was classified as inadequate in three of the four rounds of LQAS. The percentage of mothers receiving at least one dose of postpartum vitamin A changed from 54.7 to 61.3 % throughout the assessment period, however this difference was not statistically significant (*p* value = 0.44, Table 4). Although the December 2011 round of LQAS yielded the highest coverage value for post-partum vitamin A, five SAs were classified as inadequate.

Other than tetanus coverage, the findings for other vaccinations were disappointing (Table 5). From the start of the monitoring and evaluation program to the end, the coverage of pentavalent vaccine (a combination of the Haemophilus influenzae type B, Pertussis, Tetanus, Hepatitis B, and Diphtheria vaccines) did not significantly change (from 49.7 % in Round 1 to 42.1 % in Round 4, *p* value = 0.28). For pentavalent vaccine coverage, five of the seven SAs were classified as inadequate in the last assessment period. The proportion of infants receiving the measles vaccination by the first birthday also failed to meet goals set forth by Medair; the coverage significantly decreased from 72.0 to 54.1 % from the first to last survey periods (*p* value = 0.046). Notably, five SAs in May 2011, and six SAs in December 2011 were classified as inadequate. Although we collected information on the coverage of the polio vaccine, the results were difficult to interpret because the definition of the indicator was changed between the second and third rounds of LQAS to be aligned with the indicator used in the Sudan Household Health Survey. As such, the results are not included here.

**Table 5:** Immunization Indicators

Indicators	Pentavalent Vaccination by First Birthday				Measles Vaccination by First Birthday			
Assessment periods	May 2010	Oct. 2010	May 2011	Dec. 2011	May 2010	Oct. 2010	May 2011	Dec. 2011
Target Coverage	50 %	60 %	70 %	70 %	75 %	68 %	90 %	90 %
2010 Sudan Household Health Survey Result for West Darfur [28]	45.8 %				54.4 %			
Decision Rule	7	9	10	10	11	10	14	14
Average Coverage (95 % CI)	49.7 % (31.7, 67.8)	74.8 % (62.5, 84.1)	53.7 % (43.8, 63.4)	42.1 % (30.6, 54.6)	72.0 % (60.0, 81.5)	72.9 % (56.9, 84.6)	69.1 % (57.7, 78.5)	54.1 % (38.4, 69.0)
*P* value for difference between 1^st^ & 4^th^ Rounds	0.28				0.046			
Number of Successes								
SA1	10^a^	16	13	6^*^	16^a^	17	14	8^*^
SA2	^b^	14^c^	13	12	^b^	14^f^	17	13^*^
SA3	14^a^	6^*^	7^*^	11	13^a^	9^*^	8^*^	15
SA4	8	12	11	7^*^	15	13	13^*^	12^*^
SA5	12	14	9^*^	9^*d^	14	17	13^*^	11^*g^
SA6	10	12	10	7^*^	13	11	13^*^	5^*^
SA7	3^*^	10	8^*^	3^*e^	10^*^	9^*^	12^*^	6^*h^

Notes: *SAs that did not reach the benchmark. (a) 18 samples instead of the usual 19 were obtained; the decision rule is unchanged. (b): Due to insecurity, samples were not taken from SA2 in the May 2010 round of LQAS. (c) 17 samples instead of the usual 19 were obtained; the new decisiosn rule is 8. (d) 20 samples instead of the usual 19 were obtained; the new decision rule is 11. (e) 12 instead of the usual 19 were obtained; the new decision rule is 7. (f) 17 samples instead of the usual 19 were obtained; the new decision rule is 9. (g) 20 samples instead of the usual 19 were obtained; the new decision rule is 20. (h) 12 samples instead of the usual 19 were obtained; the new decision rule is 9

## Discussion

This paper has described the process of using LQAS to monitor and evaluate the primary health programs of Medair’s operations in West Darfur. Medair used the baseline LQAS findings as an integral part of strategic planning for targeted MNCH interventions and immunization efforts. Based on the results of the MNCH indicators from the first round of LQAS, Medair implemented two interventions to improve maternal and newborn care. First, Medair focused on in-service training for midwives to improve care in the immediate postpartum period for both mothers and infants. The care provided by midwives and supported by Medair was augmented with supervisory visits from advanced-practice midwives, doctors specifically trained in obstetrics and gynecology, and with biannual five to ten day in-service trainings. New strategic topics were introduced during in-service trainings, and the supervisory midwives and doctors visited various clinics to mentor midwives in the implementation of these skills and for quality improvement and quality assurance of the clinical care delivered to mothers and infants in the project area.

Second, Medair trained health promotion volunteers in communities and camps to deliver key messages about the importance of postpartum care. These messages were delivered to groups of mothers in the waiting areas at antenatal clinics and through existing women’s groups (e.g., women’s trade groups). The messages emphasized the importance of delivery with a skilled health worker; the importance of tetanus vaccines, iron supplements, and Vitamin A for the mothers; danger signs for newborns that warrant immediate medical attention; and the importance of a postpartum visit with a midwife or other health worker 2 to 3 days after giving birth and 6 weeks after the birth. Medair used data on key indicators from each round of LQAS to adjust and maximize the interventions after each round. The LQAS-based M&E system would periodically give Medair managers empirical feedback, allowing Medair to improve its programs.

Two out of the four MNCH indicators measured by the survey (protection against neonatal tetanus and births attended by a skilled health worker) showed improvement throughout the period that LQAS was used for M&E. Medair’s coverage of the two indicators was also greater than the coverage reported in the 2010 Sudan Household Health Survey for West Darfur State (Table 3). Medair’s coverage of protection against neonatal tetanus ranged from 47.2–69.7 %, while the SHHS coverage was 46.5 %. For the percentage of births attended by a skilled health professional, Medair’s coverage ranged from 34.5–52.7 %, compared with the SHHS coverage of 33.4 %. Medair’s coverage of mothers receiving a dose of postpartum vitamin A did not significantly improve. However, Medair’s coverage ranged from 45.5–61.3 % during the survey period and was higher than the 15.3 % coverage reported in the SHHS (Table 4).

The improvement in protection against neonatal tetanus and births attended by a skilled health worker is associated with the Medair managers’ decision to focus on enhancing MNCH programs in West Darfur during the 19 months that LQAS was conducted. However, the improvements were not uniform across the program areas, and Medair faced obstacles in improving MNCH indicators. For example, supervisory midwives and health promotion advisors had limited access to remote communities in SA2 during all assessment rounds due to continued armed conflict and a lack of security. Another program challenge in SAs 4, 6, and 7 was related to two human resource challenges: (1) the number of available trained midwives serving the population was insufficient for the population size, according to Sphere standards [30]; and (2) local labor laws – which necessitated specific work schedules and pay for on-call hours – resulted in interruptions in services.

Throughout the 19-month program implementation period, immunization indicators other than tetanus did not statistically improve, and in contrast to the MNCH indicators, the program coverage in the last assessment period was lower than figures reported in the 2010 SHHS (Table 5). The coverage of the Pentavalent vaccination was 42.1 % during the last assessment period, compared with a coverage of 45.8 % from the SHHS in West Darfur while Medair’s coverage of the measles vaccination was 54.1 % in the last assessment period compared with a coverage of 54.4 % from the SHHS in West Darfur. This may be due to the fact that Medair’s responsibilities were primarily supportive rather than active in West Darfur immunization activities and, although extensive, appear not to have been sufficient. Immunizations were led by the West Darfur State Ministry of Health through routine immunization programs and “mop-up” campaigns, which are campaigns in communities that go house-to-house, often as a result of a confirmed outbreak or in a push to ensure stronger coverage of a specific vaccine (e.g., polio) to immunize children who did not receive vaccines in the initial round. Medair’s supportive interventions included providing salary increases for the Expanded Program on Immunization (EPI) workers; intensifying health promotion about immunizations; providing performance-based incentives for community vaccinators; supporting the logistics for vaccines from the state capital to health facilities throughout the state (e.g., cold chain and storage); and strengthening linkages between antenatal care and immunizations areas within health facilities in the region. Changes were made to the physical layout of some clinics (e.g., placing the EPI office next to the rooms for midwifery care) to facilitate the vaccination of newborns when women came for postpartum visits. However, even with periodic data on the status of the immunization programs from LQAS, Medair’s supportive role did not allow Medair the programmatic control over administration of routine immunizations or the conduct of mop-up campaigns as was the case in other Medair programs.

Medair also faced challenges that were anticipated given working conditions in West Darfur. Supervisory Areas 6 and 7 were absorbed into Medair’s existing programs when other international NGOs were expelled in the first quarter of 2009. Medair started programs in these communities in late 2009. In the interim period, the West Darfur Ministry of Health worked to maintain the health facilities in this area, but a number of health workers resigned and several health facilities closed for a period of time. Medair’s program implementation included reopening these facilities, filling vacant health worker positions, initiating health promotion activities in the communities, and ensuring that clinical care met standards set by both Medair and the State Ministry of Health. There were also logistical challenges in the area, particularly in the rainy season, when some of the health facilities and trade centers were inaccessible due to flooding.

With these challenges, the data reflect poor performance in SAs 6 and 7. SA6 was classified as inadequate for the receipt of clean delivery kits in three of the four assessment periods, including the final round of LQAS. It was also classified as inadequate for two of the three immunization indicators in the final round. Similarly, SA7 was classified as inadequate for the coverage of measles immunization in every LQAS round and for two of the three immunization indicators in the December 2012 assessment.

Active armed conflict and the lack of security also hampered the efforts of Medair to deliver services to the catchment area and to assess the effectiveness of these services. Medair planned monthly quality assurance, supervision, and mentoring visits for clinical staff and health education volunteers, but frequently these visits were delayed due to security restrictions. When on-site visits were possible, Medair staff had limited time for these visits with local clinic staff and volunteers. At times, field visits of 3–4 h each quarter were the only times allowed for these interactions, compounding the challenges of conducting LQAS. Thus, while Medair was able to feasibly train and conduct the LQAS M&E program in a conflict setting, LQAS was a necessary but not sufficient element in translating the M&E system into improvements for Medair’s beneficiaries. LQAS by itself cannot overcome the barriers to healthcare access created by conflict and large distances between towns. If such barriers are eliminated, however, LQAS helps managers to efficiently monitor and adjust their programs.

There are limitations to the data we present. As mentioned above, accurate population estimates in Darfur are difficult to obtain; it is possible that our population estimates were inaccurate, which would affect the formulation of the sampling plan and introduce bias into our results. We did not record the number of individuals who declined to participate in the survey. Women who did participate may be systematically different than those who declined, which may also bias our results. Furthermore, during our first assessment, Medair staff was unable to enter SA2 due to armed conflict. The absence of this data in addition to the small sample sizes in LQAS in general decreases the precision of the estimates of indicators and makes drawing conclusions more difficult. As already described, access to clinics was hampered during the rainy season, and differences in indicators from one assessment period to the next six-month assessment round may have been affected by these seasonal factors. While there are other types of complex humanitarian emergencies for which LQAS may be optimal, we only examined the use of LQAS in a conflict-affected setting. Our conclusions may not apply in other types of humanitarian emergencies.

Another limitation of the data is that Medair restricted its use of LQAS to assess the population coverage of specific services related to the organization’s programs. The monitoring and evaluation system for this project focused on coverage, but did not include measurements of quality of care. Medair utilized other quality assurance measures in their supportive supervision of clinics and community health promotion volunteers. LQAS was specifically utilized to help determine the coverage of specific interventions and education initiatives that impact key health issues. Additionally, Medair needed to limit the length and time for the survey questionnaire and the limited available time of surveyors in communities. However, LQAS can be used as a tool to assess quality of care. There are multiple examples of LQAS being used for quality management [15], such as assessing the quality of care in malaria control in Nigeria [19], and the quality of health facility services in South Sudan [22]. While quality of care was not specifically measured in the presented study in West Darfur, we recommend using LQAS for this purpose in the future so that the full range of the program’s expected results (coverage and quality) can be monitored on a regular basis in a conflict-affected environment.

The next steps from the findings we present are to implement LQAS in a different complex humanitarian emergency to examine if our experience using LQAS in Darfur is consistent in other contexts. While there are published papers describing the combination of LQAS with aspects of the cluster survey design [21, 31], further investigations should also focus on comparing LQAS with the traditional cluster survey method in a complex emergency, with an emphasis on comparing the feasibility of the two survey methods to understand more clearly which method is preferable in various settings. In addition, applications of LQAS as a quality management tool in humanitarian emergencies should be further tested, including more robust efforts to support use of periodic or mid-program data to redirect activities as issues are identified. Until such studies are conducted, this paper has taken a step in illustrating how a novel method of quality assurance was adapted to provide a tool for management of a health program in an insecure, dynamic environment.

## Conclusions

The LQAS management method was beneficial for Medair’s services in West Darfur, despite the many obstacles to conducting population-level assessments of primary health programs. The surveying technique required up front coordination, time to train managers and establishment of survey and data collection techniques. After the initial training, however, local managers were able to autonomously conduct LQAS alongside their usual work, gather and analyze data, and feed the collected information back to the programs to improve service delivery in the catchment area. Program managers gained skills not only in data collection and analysis, but also in using data to make program decisions and to influence program strategy. One key learned lesson from Medair’s experience is the importance of the ability to change interventions based on results from LQAS; because Medair did not control all aspects of immunization programs in West Darfur, Medair could not fully implement changes based on the LQAS results. Nevertheless, the ability of LQAS to be easily taught to local managers and the decentralized nature of data collection and analysis in LQAS enhances its prospects for sustainability, which is vital in low-resource settings. The experience of Medair and Johns Hopkins in Sudan demonstrates that LQAS can be used in a challenging environment that is plagued by armed conflict.

## Abbreviations

EPI: Expanded Program on Immunization
IDP: Internally-displaced persons
LQAS: Lot quality assurance sampling
M&E: Monitoring and evaluation
MICS4: Multiple Indicator Cluster Survey 4
MNCH: Maternal newborn, child health
NGO: Non-governmental organization
SA: Supervisory area
SHHS: Sudan Household Health Survey
UNICEF: United Nations Children’s Fund
WFP: (United Nations) World Food Programme
